# Involvement of Astrocytes in Alzheimer’s Disease from a Neuroinflammatory and Oxidative Stress Perspective

**DOI:** 10.3389/fnmol.2017.00427

**Published:** 2017-12-19

**Authors:** Rodrigo E. González-Reyes, Mauricio O. Nava-Mesa, Karina Vargas-Sánchez, Daniel Ariza-Salamanca, Laura Mora-Muñoz

**Affiliations:** ^1^Grupo de Investigación en Neurociencias (NeURos), Escuela de Medicina y Ciencias de la Salud, Universidad del Rosario, Bogotá, Colombia; ^2^Biomedical Sciences Research Group, School of Medicine, Universidad Antonio Nariño, Bogotá, Colombia

**Keywords:** astrocytes, Alzheimer’s disease, neuroinflammation, oxidative stress, NF-κB pathway, neurodegeneration

## Abstract

Alzheimer disease (AD) is a frequent and devastating neurodegenerative disease in humans, but still no curative treatment has been developed. Although many explicative theories have been proposed, precise pathophysiological mechanisms are unknown. Due to the importance of astrocytes in brain homeostasis they have become interesting targets for the study of AD. Changes in astrocyte function have been observed in brains from individuals with AD, as well as in AD *in vitro* and *in vivo* animal models. The presence of amyloid beta (Aβ) has been shown to disrupt gliotransmission, neurotransmitter uptake, and alter calcium signaling in astrocytes. Furthermore, astrocytes express apolipoprotein E and are involved in the production, degradation and removal of Aβ. As well, changes in astrocytes that precede other pathological characteristics observed in AD, point to an early contribution of astroglia in this disease. Astrocytes participate in the inflammatory/immune responses of the central nervous system. The presence of Aβ activates different cell receptors and intracellular signaling pathways, mainly the advanced glycation end products receptor/nuclear factor kappa-light-chain-enhancer of activated B cells (NF-κB) pathway, responsible for the transcription of pro-inflammatory cytokines and chemokines in astrocytes. The release of these pro-inflammatory agents may induce cellular damage or even stimulate the production of Aβ in astrocytes. Additionally, Aβ induces the appearance of oxidative stress (OS) and production of reactive oxygen species and reactive nitrogen species in astrocytes, affecting among others, intracellular calcium levels, NADPH oxidase (NOX), NF-κB signaling, glutamate uptake (increasing the risk of excitotoxicity) and mitochondrial function. Excessive neuroinflammation and OS are observed in AD, and astrocytes seem to be involved in both. The Aβ/NF-κB interaction in astrocytes may play a central role in these inflammatory and OS changes present in AD. In this paper, we also discuss therapeutic measures highlighting the importance of astrocytes in AD pathology. Several new therapeutic approaches involving phenols (curcumin), phytoestrogens (genistein), neuroesteroids and other natural phytochemicals have been explored in astrocytes, obtaining some promising results regarding cognitive improvements and attenuation of neuroinflammation. Novel strategies comprising astrocytes and aimed to reduce OS in AD have also been proposed. These include estrogen receptor agonists (pelargonidin), Bambusae concretio Salicea, Monascin, and various antioxidatives such as resveratrol, tocotrienol, anthocyanins, and epicatechin, showing beneficial effects in AD models.

## Introduction

The loss of cognitive abilities induced by the development of dementia represents one of the main pathological burdens in humans, critically interfering with social and occupational activities. According to the World Alzheimer Report, over 46 million people live with dementia worldwide, totaling an estimated cost of US $818 billion in 2015, expecting to rise up to $1 trillion by 2018 (Prince et al., 2015). The elevated economic and social impact of dementia has been considered as a public health priority by the World Health Organization (Frankish and Horton, 2017). Many types of dementia with varied pathophysiological mechanisms have been described, but the most frequent in humans is AD accounting for 50–70% of all cases (Querfurth and LaFerla, 2010). Characteristically, AD has been divided in early-onset AD (<65 years) and late-onset AD, with the latter representing around 90% of AD-affected individuals (Mendez, 2017; Pierce et al., 2017). The development of early-onset AD has been related to an altered genetic background, explained primarily by autosomal dominant mutations in *APP* (MIM #104760), Presenilin 1 (*PSEN1*) (MIM #104311), and Presenilin 2 (*PSEN2*) (MIM #600759) genes (Lanoiselée et al., 2017). Whereas a complete explanation for the development of late-onset AD (also commonly referred to as sporadic AD) remains obscure, despite the many risk factors associated with this pathology. Among these factors are included: genetic, such as the presence of the APoE 𝜀4 allele, environmental, and several modifiable lifestyle factors (Killin et al., 2016; Van Cauwenberghe et al., 2016; Vos et al., 2017).

The main pathological hallmarks of AD are the presence of extracellular Aβ plaques, intraneuronal neurofibrillary tangles primarily composed of hyperphosphorylated tau, and brain atrophy, together with increased brain neuroinflammation (Raskin et al., 2015; Bronzuoli et al., 2016). Although many theories have been proposed to explain the pathogenesis of AD, the most widely accepted is the amyloid hypothesis, which states that Aβ dyshomeostasis is responsible for the cognitive phenotype of the disease, acting upstream and contributing to other molecular and cellular alterations observed in this condition (Selkoe and Hardy, 2016). Aβ peptide is obtained from the serial cleavage of APP, first through the action of BACE-1 also referred to as beta-secretase, and posteriorly through the gamma-secretase complex (Carroll and Li, 2016; Yan, 2017). The gamma-secretase complex, which also acts on the notch pathway, is composed of four subunits: presenilin (1 or 2), nicastrin, anterior pharynx-defective 1 (APH-1), and presenilin enhancer 2 (PEN2); with presenilin being the most actively studied and related to AD, as it contains the catalytic subunit of the complex (Ahn et al., 2010). Alterations in the cleaving process of APP produce abnormal lengthy species of the Aβ peptide which are deleterious to the brain cellular environment. These Aβ species have been reported to exhibit different profiles of toxicity, and among them, the soluble forms seem to be more neurotoxic than the fibrillary (aggregated) forms. In particular, the oligomeric form of the soluble Aβ_1-42_ is considered to be highly harmful (Wang Z.-X. et al., 2016).

The pathological study of brains from individuals with AD has revealed the presence of both neuroinflammation and OS (Lue et al., 1996; Ansari and Scheff, 2010). The precise mechanistic basis leading to the development of these changes in AD is not clear, and the debate of whether they are a causative factor or a consequence of the disease is still open. Despite the discussion, accruing evidence support a direct relationship between Aβ abnormal production and the development and/or maintenance of neuroinflammation and OS (Guerriero et al., 2016).

Plenty receptors and carriers have been reported to interact with the different presentations of Aβ, although it seems that depending on the structure of Aβ (monomer, oligomer, fibrillary), some promote clearance or degradation while others mediate the neurotoxic effects through uptake and accumulation (Jarosz-Griffiths et al., 2016). Aβ interacts and binds to several cellular-expressed pattern recognition receptors in astrocytes and microglia, initiating an innate immune response (Minter et al., 2016). Accordingly, components of innate immunity and complement cascade have been considered risk factors for the development of AD and have been associated with abnormal clearing or deposition of Aβ; in particular, variants in the genes complement receptor 1 (CR1) (Zhu et al., 2015), CD33 (Walker et al., 2015), and TREM2 (Yeh et al., 2016). Also, it has been shown that Aβ species, such as Aβ_1-42_, are able to induce the release of several proinflammatory cytokines and agents, including IL-1β, IL-6, NO, and TNFα, from glial cells (Lindberg et al., 2005; Hou et al., 2011). The precise intracellular signaling pathways involved in the proinflammatory and OS responses in neuronal and non-neuronal cells in AD are still not clear, although the NF-κB pathway has been reported to become activated in both settings (Shi et al., 2016).

Astrocytes are important CNS resident cells involved in numerous physiological aspects. Similar to neurons, astrocytes represent a heterogeneous population of cells depicting diverse functional and morphological characteristics (Ben Haim and Rowitch, 2017). Astrocytes express several markers that allow them to be distinguished from neurons and other glial cells, including GFAP, calcium-binding protein S100B, glutamine synthetase, and Aldh1L1 (Sofroniew and Vinters, 2010). Astrocytes are key for the maintenance of homeostatic balance and participate in processes such as neurotransmitter uptake and recycling, gliotransmitter release, neuroenergetics, inflammation, modulation of synaptic activity, ionic balance, and maintenance of BBB, among others (Magistretti and Allaman, 2015; Iglesias et al., 2017; Vasile et al., 2017). Precisely, due to this wide array of functional properties, astrocytes have become interesting targets for the study and treatment of numerous brain pathologies. In AD, several reports have shown that astrocytes contribute to cellular and functional degeneration, disrupting glial–neuronal and glial–vascular signaling (Acosta et al., 2017).

The aim of this paper is to review the relevant aspects concerning a possible role of astrocytes in the neuroinflammatory and OS changes observed in AD. As well, we will discuss novel neuroprotective and therapeutic measures highlighting the importance of astrocytes in AD pathology.

## Astrocytes and Alzheimer’s Disease

Different studies have shown that the cooperative activity between glia and neurons results in the modulation of cognitive functions (Perea et al., 2009; Fields et al., 2014). Neuron–glial interactions actively control synaptic plasticity and neurotransmission. The concept of “tripartite synapse” refers to this cellular network involving both presynaptic and postsynaptic neurons, as well as astrocytes (Araque et al., 1999; Perea et al., 2009). Numerous gliotransmitters released from astrocytes control synaptic plasticity in different brain structures (Yang et al., 2003; Pascual et al., 2005; Panatier et al., 2006) such as cortex (Ding et al., 2007) and hippocampus (Araque et al., 1998; Jourdain et al., 2007), and are involved in the modulation of memory and learning processes. The interruption of astrocyte’s functions and hence in glia transmission, may result in different neuropsychiatric disorders (Rajkowska et al., 1999; Cohen-Gadol et al., 2004; Fellin et al., 2004; Webster et al., 2005), as well as neurodegenerative diseases, including AD (Forman et al., 2005; Halassa et al., 2007).

### Calcium Dysregulation

A pathological increase in the amount of Aβ can induce functional and morphological changes in glial cells, including calcium dysregulation. In fact, microglia and astrocytes are activated close to senile plaques to internalize and break down Aβ (Mohamed and Posse de Chaves, 2011). This cellular activation may result in an inflammatory response and OS, playing a dual role in the pathophysiology of AD with both detrimental and neuroprotective results. Inflammatory mediators (i.e., bradykinin) may increase intracellular calcium concentration via nicotinic receptors and PI3K–Akt pathway in cultured astrocytes (Makitani et al., 2017). Tau has also been connected with astrocytes in AD, as Aβ was shown to bind the CaSR in human astrocytes, activating intracellular signaling which induced the production and release of phosphorylated Tau (Chiarini et al., 2017).

Amyloid beta has been shown to disrupt gliotransmission by enhancing calcium signaling in astrocytes (Lee et al., 2014). This calcium/gliotransmission alteration could underlie an important role of astrocytes in AD pathology. Actually, astrocytic calcium levels are abnormal in several models of AD as both acute and chronic exposure to Aβ elevates baseline calcium levels in cultured astrocytes (Haughey and Mattson, 2003; Alberdi et al., 2013; Lim et al., 2013). This calcium is partially released from intracellular sources such as the endoplasmic reticulum (Toivari et al., 2011). In addition, Aβ interacts with several types of surface receptors in astrocytes which leads to calcium entry, including purinergic receptor P2Y1 (Delekate et al., 2014), nicotinic receptors (α7-nAChRs) (Xiu et al., 2005; Lee et al., 2014), and glutamate metabotropic receptor mGluR5 (Grolla et al., 2013; Ronco et al., 2014). For instance, hippocampal astrocytes exposed to Aβ increased the frequency of NMDA receptor-mediated slow inward currents, together with calcium elevations mediated through α7nAChR activation (Pirttimaki et al., 2013). Aβ-induced dysfunction of NMDA receptors in astrocytes disrupts neuron–glial signal transmission with dramatic consequences on neuronal homeostasis, synaptic transmission, and plasticity. Therefore, neurotoxicity and selective neurodegeneration may be explained by Aβ simultaneous interaction with several receptors and neurotransmitter systems in the context of astrocyte calcium dysregulation.

### Glutamatergic Dysfunction and Excitotoxicity

In AD, it has been shown that Aβ can interrupt glutamate uptake capacity and astrocytic calcium signaling (Vincent et al., 2010; Matos et al., 2012). Also, an increase in the expression of astrocytic Tau from aged transgenic animals leads to a decline in GLT activity and therefore in subsequent neurodegeneration (Komori, 1999; Dabir et al., 2004). Some studies have demonstrated in *ex vivo* astrocyte preparations that Aβ_1-42_ decreases the expression of GLT-1 and GLAST, two major GLTs in astroglia, via adenosine A2A receptors (de Vivo et al., 2010; Matos et al., 2012). Therefore, disruption in the clearance of excitatory neurotransmitters and increased levels of Aβ and Tau from astrocytes seem to be involved in the neuronal excitotoxicity observed in AD.

Glutamate NMDA and AMPA receptors have been related to the physiopathology of AD (Parameshwaran et al., 2008). Different studies have identified the expression of functional NMDA receptors in astrocytes (Kommers et al., 2002; Lalo et al., 2006) involved in cerebral vasodilatation, synaptic transmission, and neuronal–glial signaling (Verkhratsky and Kirchhoff, 2007; Palygin et al., 2010; Parfenova et al., 2012). Hence, Aβ-induced dysfunction in the expression and function of glutamate receptors in astrocytes, mainly in NMDA receptors, can interfere with neuronal–glial communication (Mota et al., 2014). The cellular excitotoxicity produced by the excessive stimulation of NMDA receptors in neurons and astrocytes has been shown to be reduced with the use of MK801 and memantine (NMDA receptor antagonists) (Lee et al., 2010). Furthermore, due its possible therapeutic role in neurodegenerative diseases including AD, a recent antagonist (UBP141) with preferential effects on astroglial NMDA receptors has been developed (Palygin et al., 2010, 2011). A better comprehension of the differences between neuronal and glial NMDA receptors may provide key elements for the development of novel therapeutics which primarily or selectively target astrocytic function. As well, Aβ can induce glutamate release from astrocytes resulting in an extrasynaptic activation of NMDA receptors. In this case, nitromemantine, which selectively inhibits extrasynaptic NMDA receptors, may protect against Aβ-induced synaptic dysfunction in the hippocampus (Talantova et al., 2013). Additionally, nitromemantine may prevent the synapse-destroying effects of Aβ/α7-nAChR signaling (Dal Prà et al., 2015).

Therefore, using astrocytic signaling as a possible target for drug development may have a therapeutic function in AD’s prevention and control. The antiepileptic drug levetiracetam has shown to reverse synaptic dysfunction as well as memory and learning deficits in human APP (hAPP) transgenic mice (Sanchez et al., 2012). Moreover, a retrospective observational study has shown clinical benefits of levetiracetam in early AD (Vossel et al., 2013). One way this drug may act is increasing glutamate and GABA transporters in astrocytes (Ueda et al., 2007). Chronic administration of levetiracetam may attenuate glutamate excitotoxicity and increase inhibitory neurotransmission. This molecular mechanism involving astrocytes may result in a reduction of cognitive abnormalities in AD.

### Aβ Clearance

Astrocytes also participate in the degradation and removal of Aβ as they express different types of proteases involved in the enzymatic cleaving of Aβ. The metalloendopeptidases NEP, IDE, and ECE1 and ECE2 have been reported to be expressed in astrocytes, and are involved in the degradation of monomeric Aβ species (although NEP also hydrolyze oligomeric forms) (Mulder et al., 2012; Ries and Sastre, 2016). It has been proposed that the modification from “natively folded-active” to “aggregated-inactive” form of IDE and NEP may be a relevant pathological mechanism in late-onset AD (Dorfman et al., 2010). Astrocytes also express and secrete several MMPs, including MMP-2 and MMP-9, which degrade both monomeric and fibrillar extracellular forms of Aβ (Ries and Sastre, 2016). Furthermore, it was found in APP/presenilin 1 transgenic mice that astrocytes surrounding Aβ plaques increased the expression of both MMP-2 and MMP-9 (Yan et al., 2006; Yin et al., 2006).

Apolipoprotein E is primarily produced by astrocytes in the CNS and has been proposed to play a major role in AD. In mice, ApoE(^-/-^) astrocytes have been shown to fail to respond or internalize Aβ deposits to the same extent as do wild-type astrocytes (Koistinaho et al., 2004). As well, mice astrocytes expressing the ApoE 𝜀4 allele were less effective eliminating Aβ plaques than those astrocytes expressing the ApoE 𝜀3 allele (Simonovitch et al., 2016). Astrocytes derived from human induced pluripotent stem cells (iPSC) which expressed the ApoE 𝜀4 allele failed to support neuronal neurotrophic functions such as survival and synaptogenesis (Zhao et al., 2017). As the presence of ApoE 𝜀4 allele is considered a major risk factor in AD while the presence of ApoE 𝜀2 allele is considered a protective factor, a differential regulation of these isoforms regarding the presence of Aβ and associated responses such as neuroinflammation has been proposed (Dorey et al., 2017).

### AD and Astrocyte Imaging

One of the most important research areas in AD is related to the development of biomarkers. Although several types of biomarkers have been explored, there is still not one that specifically diagnose, differentiate, and predict the rate of decline between populations of cognitively healthy/preclinical dementia, mild cognitive impaired and AD individuals (Fiandaca et al., 2014; Salvatore et al., 2015; Huynh and Mohan, 2017). Due to their fundamental role in CNS homeostasis, astrocytes could be considered as possible targets for tracking and studying *in vivo* changes in AD as well as serving as a biomarker for the disease.

L-Deprenyl is a selective inhibitor of the enzyme MAO-B, predominantly found on astrocytes (Levitt et al., 1982). This compound has been successfully used *in vitro* and *in vivo* to study the distribution and activity of MAO-B through different techniques including quantitative autoradiography and positron emission tomography (PET) (Kumlien et al., 1992; Arakawa et al., 2017). In postmortem samples from individuals with AD, the activity of both MAO-B and the binding of [3H]L-deprenyl was found to be increased in many brain regions (Jossan et al., 1991). In addition, MAO-B has been found to be increased during reactive astrocytosis in neurodegenerative conditions (Ekblom et al., 1993, 1994). As astrocytes produce MAO-B and this enzyme is increased during reactive astrocytosis, which is a process observed in AD, it seemed plausible to use L-dyprenyl as a marker of astrocytosis in this condition.

Accruing evidence seems to support the use of L-deprenyl in AD. A comparative study using PET between one of the currently accepted biomarkers for AD, the 11C-Pittsburgh Compound B, and (11)C-deuterium-L-deprenyl, concluded that the latter provided non-redundant information on both functional and pathologic aspects of the disease (Rodriguez-Vieitez et al., 2016a). Furthermore, L-deprenyl has provided valuable information about the stage of progression of AD. In a human study, the highest binding for (11)C-deuterium-L-deprenyl was observed in Braak I–II (initial AD stages), whereas it decreased with the most advanced Braak stages (Gulyás et al., 2011). Similar results have been obtained in other studies using (11)C-deuterium-L-deprenyl, where astrocytosis is prominent at the initial phases, even at preclinical stages, and then declines as the disease progresses (Carter et al., 2012; Schöll et al., 2015; Rodriguez-Vieitez et al., 2016b). In addition, a similar laminar binding pattern for tau and [3H]L-deprenyl at the temporal lobe was recently demonstrated, suggesting tau deposits and astrocytic inflammatory processes are closely related in AD (Lemoine et al., 2017). All these results point to an early contribution of astrocytes in AD pathology.

### GABA–Glutamine Cycle

Neurons and astrocytes work in a coordinated way throughout different metabolic pathways to synthesize and release glutamate and GABA (Bak et al., 2006). At inhibitory synapses this pathway is called the GABA–glutamine cycle and it depends on GABA transporters and a multi-enzyme machinery that coordinates this process (i.e., GABA transaminase, glutamate decarboxylase, and glutamine synthetase) (Bak et al., 2006; Hertz, 2013). In AD, the processes related to GABA–glutamine cycle and GABA release from astrocytes seem to be altered. The glutamine–glutamate/GABA cycle consists of the transfer of glutamine from astrocytes to glutamatergic and GABAergic neurons. This process depends on glutamine synthetase and the tricarboxylic acid cycle (Walls et al., 2015). A reduction in pyruvate carboxylation, glutamine levels, and tricarboxylic acid cycle turnover in GABAergic neurons and astrocytes was shown in the transgenic rat AD model, McGill-R-Thy1-APP (Nilsen et al., 2014). Similarly, reduced expression of glutamine synthetase in postmortem AD brain samples indicates a profound alteration in neurotransmitter and protein synthesis, as well as metabolic dysfunction (Robinson, 2000). Astrocytes may produce and release GABA, with a main role on hippocampal synaptic plasticity function during memory processing. Increased activity of glutamate decarboxylase enzyme was found in glial synaptosomes obtained from the cortex of APP/TS1 transgenic mice, suggesting Aβ plaques stimulate GABA synthesis from astrocytes (Mitew et al., 2013). Reactive astrocytes from APP/PS1 transgenic mice have also been shown to produce GABA involving MAO-B, and release it through the bestrophin 1 channel, in an aberrant manner (Jo et al., 2014). In the same study, the suppression of GABA production or release from astrocytes completely restored the cognitive deficits and impairments in synaptic plasticity observed in the mice. Under physiological conditions, astrocytic GABA exerts a disinhibitory action at the perforant path to dentate gyrus neurons via GABA_B_ receptors on interneurons. However, in the APPswe/PSEN1dE9 mice, it has been shown an inhibitory action of astrocytic GABA by targeting GABA_A_ receptors in glutamatergic terminals (Yarishkin et al., 2015). These results provide a useful specific GABAergic target aimed at memory impairment reduction in AD. Alterations in the metabolic functions of astrocytes and consequently in glutamate and GABA–glutamine cycles may help explain cognitive disorders in AD (Le Prince et al., 1995; Robinson, 2000; Nilsen et al., 2014). Neurotransmitter transporters and effectors together with GABA-metabolizing enzymes are of special interest in drug development regarding therapeutical options for GABA-related neurological dysfunctions such as AD (Sarup et al., 2003; Nava-Mesa et al., 2014; Mutis et al., 2017; Sánchez-Rodríguez et al., 2017). Although special attention should be taken regarding the differential functional roles of neuronal and glial neurotransmitter transporters and overlying GABA/glutamate metabolic pathways in the development of high selective cell-specific drugs, in order to avert pharmacological interactions and unexpected side effects.

### Metabolic Compromise

The metabolic cooperation between astrocytes and neurons is essential to the brain functioning. The energy metabolism of neurons depends on blood oxygen supply but also on astrocytic glucose transporters, mainly GLUT1 (Morgello et al., 1995). In addition, astrocytes may convert glycogen to lactate during periods of higher activity of the nervous system (Falkowska et al., 2015). Both *in vivo* and *in vitro* studies indicate that astrocytes participate in the regulation of cerebral blood flow according to neuronal activity and metabolic demand (Magistretti and Pellerin, 1999; Magistretti, 2006). Therefore, astrocytes are key to guarantee an adequate coupling between brain activity and metabolic supply. Several studies have shown reduced cerebral glucose metabolism in early stages of AD and correlation with symptoms severity (Desgranges et al., 1998; Mosconi et al., 2005, 2006, 2008). As mentioned early, Aβ affects neuronal excitability and it also may reduce astrocytic glycolytic capacity (Soucek et al., 2003; Schubert et al., 2009) and reduce the neurovascular unit function (Acosta et al., 2017; Kisler et al., 2017). Moreover, reductions in GLUT1 and lactate transporters in astrocyte cultures derived from transgenic AD mice have been reported (Merlini et al., 2011). In AD, the resulting metabolic dysfunction may alter the overall oxidative neuronal microenvironment (Mosconi et al., 2008). The chronic sustained effect of diminished lactate supply, increased neuronal activity, and reduced neurovascular coupling, underlines the OS increase during AD. Therefore, astrocytes are crucial players either acting as protectors against OS or participating in the progression of AD. The specific role of astrocytes on inflammatory response and OS damage will be reviewed in the next sections.

## Neuroinflammation, Alzheimer’s Disease, and Astrocytes

Inflammation is a protective physiological response necessary to regulate processes associated with damage mechanisms in the organism. Several actions related to general inflammatory activities include protection against microorganisms, tissue repair, and removal of cellular debris. The CNS possesses some characteristics that differentiate the immune and inflammatory activities of the brain and spinal cord from those occurring in the rest of the body. Mainly, these differences arise through the presence of the BBB, which restricts the pass of leukocytes into the brain parenchyma, and also due to the cellular interactions of microglia and astrocytes, responsible for most of the immune/inflammatory CNS responses (Ransohoff et al., 2015). Although neuroinflammation arises innately as a protective mechanism when injury is present in the CNS, alteration in any of the components of this response may compromise the cellular microenvironment and become noxious to the brain. Many neurodegenerative conditions, including AD, have been associated with the presence of abnormal neuroinflammation (Ransohoff, 2016).

The role of astrocytes in neuroinflammation has been highlighted in the past years with many observations both *in vivo* and *in vitro* depicting the importance of these glial cells in this process (Colombo and Farina, 2016). In fact, an increase in the expression of GFAP is commonly considered as a hallmark of neuroinflammation in many neurodegenerative conditions, including AD (Millington et al., 2014). Astrocytes, together with microglia, react to a diverse range of pro- and anti-inflammatory agents (Sofroniew, 2014). Depending on the cytokine, astrocytes modify their phenotype to either activated or deactivated state. Increased levels of INF-γ, IL-1β, IL-6, and TNFα induce astrocytes to adopt a classical activation state (increased activation of NF-κB pathway, production of ROS and NO, and release of IL-1β, IL-6, and TNFα), while increased levels of IL-4 and IL-13 induce an alternative activation (increased secretion of IL-4 and decreased production of ROS and NO); oppositely, high levels of IL-10 and TGF-β induce astrocytic deactivation (reduced immune surveillance and proinflammatory signaling) (Dá Mesquita et al., 2016).

Furthermore, the reactive state of astrocytes may also depend on the source of injury (neuroinflammation or ischemia), indicating the complex range of responses these cells are capable to produce (Zamanian et al., 2012). In a recent paper, a new classification of reactive astrocytes was proposed, designating A1 those astrocytes that developed a neurotoxic phenotype and A2 those that depicted neurotrophic and neuroprotective characteristics (Liddelow et al., 2017). The authors also reported that the presence of IL-1α, TNFα, and C1q (all three released from microglia) promoted the appearance of A1 astrocytes and that this phenotype was found to be predominant in brain tissue from AD patients. These findings raise a number of questions regarding the manner in which the brain deals with different types of injuries and specifically how astrocytes and astrocytic-cellular interactions induce either a protective or harmful profile. An increase in A1 astrocytes seems to occur in AD, but still is not clear if Aβ induces this phenotype or if another specific agent is involved in this reaction. Nonetheless, it has been reported that the interaction of Aβ with astrocytes induces a pro-inflammatory profile and even astrogliosis (Batarseh et al., 2016).

### RAGE, Astrocytes, and Amyloid Beta

Amyloid beta has been reported to interact with numerous cellular receptors and astrocytes express a large amount of them, including TLRs, scavenger receptors, glycoprotein receptors, lipoprotein receptors, RAGE, acetylcholine receptors, complement and chemokine receptors, T-cell receptors, and mannose receptor, among others (Farfara et al., 2008). Binding of Aβ to different types of receptors seems to depend on the Aβ peptide form (monomer or fibrillar). For example, IR- and SEC-R bind monomeric forms of Aβ, scavenger receptor CD36 and glycoprotein receptors lactadherin, and CD47 prefers fibrillary Aβ, while RAGE, ApoE, and nAChR α7nAChR bind both monomer and fibrillar forms (Verdier et al., 2004). The specific outcome of all these complex Aβ-astrocytic interactions is still under research, as the precise intracellular and intercellular communication changes prompted by the different types of Aβ acting on these receptors is yet to be elucidated. Despite the gaps in knowledge, the activation of some receptors, in particular RAGE, has been reported to induce proinflammatory changes in astrocytes when exposed to Aβ (González-Reyes and Graciela Rubiano, 2016).

Advanced glycation end products receptor is a multiligand pattern-recognition receptor, member of the immunoglobulin superfamily with a variety of isoforms present in brain cells (Ding and Keller, 2005). In addition to Aβ and several AGE, RAGE can bind DNA-binding protein HMGB1/amphoterin (Hori et al., 1995) and S100/calgranulins (Hofmann et al., 1999). The main intracellular pathway activated through RAGE is the NF-κB pathway (Tóbon-Velasco et al., 2014), although it can also activate other downstream pathways including Cdc42-Rac, p21ras and MAPK, JNK, and ERK (González-Reyes and Graciela Rubiano, 2016). Furthermore, astrocytes have been reported to adopt a phagocytic profile capable of engulfing Aβ, mediated by CD36, CD47, and RAGE receptors (Jones et al., 2013). Apart from Aβ, the interaction of astrocytic RAGE with other ligands, such as S100B, may also be involved in AD neuroinflammation (Cirillo et al., 2015). These findings point to the interaction of RAGE/NF-κB pathway in astrocytes as an important factor in the development or maintenance of inflammation in AD.

### Astrocytes and NF-κB Pathway

The transcription factor NF-κB is currently considered as an important agent related to neuroinflammation in AD (Shi et al., 2016). NF-κB is known to be mainly activated by two pathways, the canonical (or classical) and the non-canonical (or alternative) (Nakajima and Kitamura, 2013). The canonical pathway involves activation of various receptors including RAGE and cytokine receptors, such as TNF receptor, IL-1 receptor, and the TLR family. These will induce the further activation of many downstream agents, in special IKKs alpha (IKKα) and beta (IKKβ), and NEMO (in charge of the degradation of the cytoplasmic inhibitor IKBα), and the subsequent complexes (mainly RelA) that act as transcription factors in the nucleus (Marcu et al., 2010; Wan and Lenardo, 2010). The non-canonical pathway, also known as the NEMO-NF-κB-independent pathway, occurs when NF-κB is activated by specific recruitment of TRAF2 and TRAF3, and involves p52 and RelB (Morgan and Liu, 2011). Both canonical (Wang et al., 2013) and non-canonical (Akama and Van Eldik, 2000) activation of NF-κB has been observed in astrocytes stimulated with Aβ, but still is not clear which type predominates in AD or if a differential NF-κB activation is related to the stage of the disease. It has been reported that most of the cytokines and chemokines produced by non-stimulated and activated astrocytes are direct targets of the NF-κB pathway, suggesting a central role of this factor in the proinflammatory (neurotoxic) and immunoregulatory (neuroprotective) actions of astrocytes in the CNS (Choi et al., 2014). Also, NF-κB is involved in other functions such as neuronal survival, differentiation, apoptosis, neurite outgrowth, and synaptic plasticity, all found to be altered in AD (Mémet, 2006).

In astrocytes and microglia, the activation of NF-κB due to Aβ stimulation leads to the production of the pro-inflammatory cytokines IL-1β, IL-6, iNOS, and TNFα (Bales et al., 1998; Akama and Van Eldik, 2000; Hou et al., 2011). In rats treated with Aβ_1-42_ oligomers, it was shown that COX-2, IL-1β, and TNFα were expressed in reactive astrocytes surrounding the Aβ-injection site and in nearby blood vessels, as well was found co-localization of NF-κB proteins with GFAP and COX-2 (Carrero et al., 2012). In primary astrocytic and mixed astrocytic-neuronal cell cultures from rats, the use of minocycline, an anti-inflammatory agent, reduced astrocytic inflammatory responses together with a decrease in neuronal loss, caspase-3 activation, and caspase-3-truncated Tau species in neurons (Garwood et al., 2011). Minocycline has been shown to inhibit the NF-κB signaling pathway in spinal rat astrocytes (Song et al., 2016). Other reports of beneficial outcomes due to regulation of the NF-κB signaling pathway in astrocytes were reviewed by Colombo and Farina (2016). Although an exaggerated neuroinflammatory response is observed in AD, an absolute suppression of the NF-κB signaling pathway may be undesirable and even worsen the pathological condition. In APPswe/PS1dE9 transgenic mice, the suppression of NF-κB attenuated astrogliosis in the hippocampus and cortex of the animals but increased the amount of Aβ_1-42_, suggesting a role of astrocytic-mediated neuroinflammation in the clearance of Aβ (Zhang et al., 2009). As well, the clinical evidence for the use of non-steroidal anti-inflammatory drugs (NSAIDS) in AD patients has not proven to be of benefit (Aisen et al., 2008; Szekely et al., 2008; Beeri et al., 2012; Alzheimer’s Disease Anti-inflammatory Prevention Trial Research Group, 2013).

### Inflammatory Induction of Aβ in Astrocytes

Astrocytes not only are activated and induced to release chemokines and cytokines in the presence of Aβ, these cells also react to the presence of pro-inflammatory cytokines and even increase the production of Aβ in response. Therefore, the presence of inflammation is capable of increasing the production of Aβ. In addition, the development of neuroinflammation has also been related to cognitive changes in AD (Westin et al., 2012; Echeverria et al., 2016; Laurent et al., 2017).

Neuroinflammation in AD is characterized by the accumulation of cytokines such as IL-1β, IL-6, TNF-α, or TGF-β, which can contribute with cerebral amyloid deposition, augmentation of APP expression, Aβ formation, and subsequent recruitment and activation of microglial cells (Esler and Wolfe, 2001). In general, TNF-α, IL-1β, IFN-γ, L-6, and TGF-β are able to stimulate β-secretase and γ-secretase enzymatic activity through a JNK-dependent MAPK pathway, which cleaves APP and initiates Aβ formation (Liao et al., 2004). Astrocytes express and respond to a large scope of cytokines and chemokines suggesting a central role in the inflammatory-induced production of Aβ.

A study reported that a systemic immune challenge in wild-type mice during late gestation induced the development of AD-like pathology during aging, with animals displaying increased levels of hippocampal APP and altered Tau phosphorylation, together with microglia and astrocytic activation (Krstic et al., 2012). Additionally, it was shown that LPS-induced systemic inflammation in mice could contribute to cognitive impairment and increased expression of APP and Aβ_1-42_, associated with increased production of inflammatory mediators such as COX-2, IL-1, and iNOS (Lee et al., 2008). The same paper reported that these changes were accompanied with astrocytic activation. Other studies have found evidence, both in human and murine models, that inflammation induces the expression of Aβ. Primary astrocytes from mice, treated with a combination of TNFα and INF-γ, significantly increased levels of BACE1, APP, and Aβ_1-40_ (Zhao et al., 2011). In human primary astrocytes, treatment with INF-γ in combination with either TNFα or IL-1β induced the secretion of Aβ_1-40_ and Aβ_1-42_ (Blasko et al., 2000). Furthermore, TGF-β1 was found to induce overexpression of APP in astrocytes but not in neurons (Lesné et al., 2003). Cytokines seem to act on the 5′-untranslated region (5′-UTR) of the APP gene in astrocytes (Lahiri et al., 2003).

On the one hand, it seems that the presence of Aβ is able to induce the production and release of pro-inflammatory cytokines and chemokines from astrocytes, which could as well act in an autocrine manner to further induce the production of Aβ from astrocytes and possibly other cells. On the other hand, these results suggest that inflammation may be present at early stages (pre-clinical) of the disease or even that inflammation may be responsible for the appearance of pathological Aβ production and accumulation. Under both circumstances, astrocytes appear to be deeply involved in inflammatory changes observed in AD.

### Astrocytes and Other Mechanisms of Neuroinflammation

Other factors related to astrocytes may contribute to the appearance or enhancement of neuroinflammation in AD. For example, the presence of elevated glucose levels (as found in diabetes) has been shown to increase neurotoxicity and the release of pro-inflammatory cytokines from primary human astrocytes (Bahniwal et al., 2017). A relation between AD and diabetes/metabolic syndrome has been explored previously (González-Reyes et al., 2016), as both Aβ and AGE bind to RAGE in astrocytes. As well, pro-inflammatory signaling in astrocytes may involve changes in the expression of the calcium-dependent phosphatase CaN, which has been shown to interact with another transcription factor involved in inflammatory responses, the NFAT (Acosta et al., 2017). Enhanced nuclear accumulation of CaN/NFAT was observed in human AD hippocampus and astrocytic cultures treated with Aβ (Abdul et al., 2009). In addition, it has been reported that Aβ deregulates calcium homeostasis via CaN and its downstream target NF-κB, as well as increasing NF-κB-dependent expression of mGluR5 and IP3R2 in astrocytes (Lim et al., 2013). Changes in mGluR5 and IP3 receptor expression have been reported in astrocytes surrounding amyloid plaques in a genetic mouse model of AD (Norris et al., 2005). Another possible factor contributing to the presence of neuroinflammation is the BBB, as in AD, it has been reported that the BBB occasionally loses its integrity (Chakraborty et al., 2017). This may be explained in part thanks to the accumulation of Aβ in brain blood vessels and also due to the associated vascular inflammation, allowing crossed communication between the peripheral immune system and the brain (Takeda et al., 2014). As astrocytes have a very important interaction with the BBB and its functional components are plausible to consider their involvement in BBB-associated neuroinflammatory changes in AD.

Uncontrolled neuroinflammation is a critical element in the progression of AD, impairing the normal function of the CNS. Astrocytes, together with microglia, are the main cells involved in the inflammation/immune responses of the CNS. The presence of Aβ activates different astrocytic cell receptors, mainly RAGE, inducing the activation of the inflammatory pathway NF-κB responsible for the transcription of numerous pro-inflammatory cytokines and chemokines in astrocytes. In addition, the presence of pro-inflammatory cytokines such as IL-1β can act on astrocytes stimulating the production of Aβ and perpetuating a pro-inflammatory profile in astrocytes. Astrocytes are key in the maintenance of the homeostatic balance of the CNS and use the mechanism of reactive astrogliosis as a defensive reaction (Pekny et al., 2016), therefore is fundamental to understand the pathophysiological process that causes astrocytes to convert from a protective agent into a cell that produces a maladaptive astrogliosis response in AD (**Table 1**).

**Table 1:** Summary of studies reporting effects of Aβ or AD on pro-inflammatory and anti-inflammatory cytokines and chemokines in astrocytes.

Experimental model	Pro-inflammatory agents	Anti-inflammatory agents	Reference
Primary culture of rat astrocytes treated with Aβ_1-42_	↑TNFα; ↑IL-1β; ↑IL-6; ↑COX-2; ↑PGE2; ↑IL-17; ↑INF-γ; ↑IP-10	↑IL-13	Hu et al., 1998; Akama and Van Eldik, 2000; Garwood et al., 2011; Wang et al., 2013; Aguirre-Rueda et al., 2015
Isolated rat astrocytes treated with Aβ_25-35_	↑TNFα; ↑IL-1β		Del Bo et al., 1995; Ayasolla et al., 2004
Primary cultures of rat astrocytes treated with Aβ_1-40_	↑IL-1β; ↑IL-6		Bales et al., 1998
Mixed cultures of rat neurons and astrocytes treated with Aβ_1-42_ and Aβ_25-35_	↑TNFα		Masilamoni et al., 2005b
Primary cultures of rat astrocytes treated with spherical aggregates of synthetic Aβ	↑IL-1β		Todd and Garrard, 1979
*In vivo* i.c. infusion of Aβ_1-42_ oligomers in rats	↑IL-1β; ↑TNFα; ↑COX-2		Carrero et al., 2012
Primary culture of mice astrocytes treated with Aβ_1-42_	↑IL-1β; ↑IL-6; ↑TNFα	↓TGF-β1	Liu et al., 2009; Hou et al., 2011
Brain sections of Tg2576 mice stained for GFAP	↑IL-1β; ↑IL-6; ↑INF-γ; ↑IL-12	↑TGF- β1; ↑IL-10	Mehlhorn et al., 2000; Apelt and Schliebs, 2001; Abbas et al., 2002
*In vivo* i.c.v infusion of Aβ_1-42_ oligomers in mice		↓TGF-β1	Diniz et al., 2017
*In vivo* i.c.v infusion of Aβ_1-42_ in IL-32β transgenic mice	↑IP-10; ↑GM-CSF	↑IL-13	Yun et al., 2015
Astrocytoma human cell line U-373 MG treated with Aβ_1-40_ (co-treated with IL-1β)	↑IL-6		Gitter et al., 1995
Postmortem brain tissue from patients with AD, stained for GFAP (or other astrocytic marker)	↑IL-1β; ↑IL-6; ↑IL-18		Ojala et al., 2009; Bouvier et al., 2016

*TNFα, tumor necrosis factor alpha; TGF-β1, transforming growth factor beta 1; COX-2, cyclooxygenase-2; PGE2, prostaglandin E2; GFAP, glial fibrillary acidic protein; IP-10, interferon gamma-induced protein 10; GM-CSF, granulocyte–macrophage colony-stimulating factor; i.c., intracerebral; i.c.v., intracerebroventricular.*

## Oxidative Stress, Alzheimer’s Disease, and Astrocytes

Oxidative stress is the result of a dysregulation between the amount of free and non-free radicals produced, including ROS and RNS. This can be attributed to the loss of homeostasis due to mitochondrial overproduction of oxidants over the production of antioxidants (Swomley and Butterfield, 2015). Among the most important ROS are the peroxyl radicals (ROO⋅), NO, the superoxide radical anion ($O_{2}^{–}$), the hydroxyl radical OH⋅ and some other non-radical species such as peroxynitrite (ONOO^-^), single oxygen (O_2_), and hydrogen peroxide (H_2_O_2_) (Dasuri et al., 2013). ROS, as well as RNS, are produced under physiological conditions during the common metabolic pathways. These reactive species act on second messengers and subsequently may influence several signaling pathways in a positive or negative form, depending on the regulatory mechanism of its concentration, called redox regulation (Valko et al., 2007). Likewise, mitochondria are able to produce antioxidants which counteract the harmful effects of OS to maintain the balance between the production and detoxification of ROS. These antioxidants are classified in two main groups: enzymatic antioxidants such as SOD, catalase, antioxidant GSH, GPX, GSH reductase, and GSH-*S*-transferase, and non-enzymatic antioxidants such as GSH, thioredoxin, vitamins A, E, and C, flavonoids, and proteins like albumin and metallothionein (Valko et al., 2007; Halliwell, 2012).

### Oxidative Stress and Alzheimer’s Disease

The development of OS in AD has been related to mitochondrial dysfunction, leading to superoxide overproduction ending in synaptic damage (Friedland-Leuner et al., 2014; Bhat et al., 2015). Mitochondrial dysfunction in AD seems to be linked to the increased presence of ROS and RNS (Islam, 2017). Müller et al. (2010) observed a decreased mitochondrial potential in transgenic Thy1-APP751SL mice. The same authors reported that increased intracellular Aβ production might trigger mitochondrial dysfunction quite early and independently of Aβ plaques and, that the accumulation of these alterations with aging lead to disruption of respiratory chain complexes (mainly III and IV) and significant reduction in the generation of NADH. The authors suggested that progressive increase in oxidant production together with a decrease in antioxidant components may conduce to the loss of brain homeostasis observed in AD. However, in AD, it has been demonstrated that prior to the appearance of senile plaques, brains present glucose hypometabolism due to abnormal oxidative metabolic routes in the mitochondria, which also induce increased ROS production and subsequent oxidative cell damage (Maruszak and Żekanowski, 2011). Additionally, variants of gene expression profiles in AD have shown downregulated expression of NeuroD6, which encodes a transcription factor involved in triggering antioxidant responses and the maintenance of the production of mitochondrial antioxidants (Uittenbogaard et al., 2010; Fowler et al., 2015). Also, RNA-Seq and microarray data analysis indicated a consistent downregulation of NeuroD6 in brains of individuals with AD, suggesting downregulation of NeuroD6 as a possible biomarker for AD (Satoh et al., 2014).

The role of ROS and OS in neurodegenerative diseases, including AD, is not entirely clear, although it has been observed that a modest level of oxidative RNA damage occurs during the process of aging in brain neurons, but a prominent level of oxidative RNA damage is present in vulnerable neurons which correspond to the earliest stage of cognitive decline in the transition from cognitively normal aging to AD (Nunomura et al., 2012). Furthermore, DNA bases are vulnerable to OS damage involving hydroxylation, protein carbonylation, and nitration in AD (García-Blanco et al., 2017). Changes in oxidative markers have been reported in brain regions such as hippocampus and inferior parietal cortex, which are also compromised in AD (Floyd and Hensley, 2002).

Brain is considered to be especially vulnerable to OS and susceptible to lipid peroxidation because of its high lipid and poly-unsaturated fatty acids content, and its low concentrations of antioxidants (Butterfield et al., 2013). In AD, neurotoxic effects of Aβ induce OS through lipid peroxidation, protein degradation, and amino acid oxidation, which in turn increase the production of ROS and RNS by positive feedback (Swomley and Butterfield, 2015). Alkenals, 4-hydroxynonenal (HNE), and 2-propenal (acrolein) are reactive products obtained from lipid peroxidation induced by Aβ. These agents can modify covalently some amino acids residues or change protein conformation, which in turn affects its function. Thus, a coupling between increased lipid peroxidation and structural modification of GLT-1 has been proposed, explained by increased HNE binding due to excessive Aβ_1-42_ (Butterfield et al., 2002). These events can compromise astrocyte function, inducing glutamate transport inhibition and increasing excitotoxicity to neurons in AD.

### Astroglia Role in Oxidative Stress

Astrocytes seem to be involved in the processes leading to the appearance or maintenance of OS in AD. Abramov et al. (2004a), working in rat astrocytes, have shown that Aβ treatment increases intracellular-free calcium influx from the extracellular space, and induces changes in mitochondrial functions. These changes are associated with the activation of NOX due to Aβ interaction on the membrane, which, in turn, induces [Ca^2+^]i changes. The main changes leading to mitochondrial dysfunction in astrocytes are associated with mitochondrial depolarization, increased conductance, and presence of mitochondrial permeability transition pores (Duchen, 2000). A possible mechanism which explains why calcium influx is induced by Aβ into astrocytes could be related with the formation of calcium selective channels on the membrane, which seem capable of generating a different conductance (Abramov et al., 2004b). These channels have been shown to be formed by insertion of Aβ peptides in the membrane, and also are arranged in a structural configuration which requires a lesser content of cholesterol on the lipidic membrane (Kawahara and Kuroda, 2001; Arispe and Doh, 2002; Arispe et al., 2007).

Additionally, it has been found that Aβ_1-42_ oligomers are key factors on the induction of OS stress by astrocytes. Aβ_1-42_ oligomers binding to RAGE on astrocytes induce ROS production via NOX complex activation (Askarova et al., 2011). However, astrocytes are also able to trigger ERK1/2 pathways and cytosolic phospholipase A2 phosphorylation, independent of NOX activation, which in turn causes mitochondrial dysfunction by decreasing mitochondrial membrane potential, enhancement of NOX activity, and overproduction of ROS (Zhu et al., 2006). Astrocytes seem to be a primary target of Aβ, as this peptide induces various effects related to OS such as altered intracellular calcium signaling and calcium-dependent reduction in astrocytic GSH (Abramov et al., 2004b). Although this GSH depletion affects astrocytes, neurons are the cells that show a higher rate of cell death, suggesting that neurotoxicity reflects the neuronal dependence on astrocytes for antioxidant support. This could better be explained by the fact that astrocytes are the producers of the primary elements required for the production of GSH in neurons, such as glycine and cysteine (Abramov et al., 2003; Gandhi and Abramov, 2012). Regarding GSH, it has also been shown that in cultured astrocytes, a prolonged incubation with Aβ reduces induction of the transporter ABCC1 which is the main pathway for GSH release (Ye et al., 2015). Astrocytes play a central role in maintaining the neuronal integrity, nevertheless cytokines and neurotransmitters released by damaged or activated astrocytes may increase the neurotoxicity and vulnerability of neurons. During chronic OS, as observed in AD, the crosstalk communication between astrocytes and neurons is impaired resulting in disrupted memory consolidation. This compromise in memory formation is probably due to calcium overload and activation of MAPK pathways in astrocytes, which involve as well the JNK/SAPK pathways, and may conduce to anomalous deleterious signaling, including autophagic astroglial signals and apoptosis (Ishii et al., 2017).

### Oxidative Stress and the Connection with Neuroinflammation

During neuroinflammation, increased concentrations of ROS/RNS may lead to the activation of the transcription factor NF-κB which induces the overexpression of NO synthases in astrocytes and microglia, in particular NOX2 and iNOS, resulting in peroxynitrite production by superoxide and NO reaction producing neuronal damage (Saha and Pahan, 2006; Brown, 2007; Morgan and Liu, 2011). Moreover, NF-κB activation induces the expression of COX-2 and cytosolic phospholipase A2, which in turn stimulate the generation of prostaglandins, promoting inflammation and OS (Hsieh and Yang, 2013). Castegna et al. (2003) reported that the formation of peroxynitrite ONOO^-^ leads to protein nitration in enzymes, such as alpha and gamma enolases, implicated in brain glucose metabolism. Thus, the signaling pathway NF-κB, which is also heavily involved in inflammatory reactions, has been proposed to be involved in OS, as a direct crosstalk between ROS and NF-κB has been reported (Turillazzi et al., 2016). In AD, the presence of chronic OS alters the protective physiological role of the NF-κB transcription pathway, which normally promotes cell survival and prevents apoptosis and necrosis, through modulation of the JNK signaling pathway (Morgan and Liu, 2011). In astrocytes, it was reported that under certain conditions, IL-1β may act stimulating astrocytic GSH production, and potentially, augmenting total antioxidant capacity in the brain, via an NF-κB-dependent process (He et al., 2015). In this way, NF-κB pathway has been associated with both pro-oxidant and antioxidant roles. In AD, an alteration of this pro- and anti-oxidant role of NF-κB in astrocytes seems to be present, tending toward a pervasive pro-oxidative and pro-inflammatory profile (**Table 2**).

**Table 2:** Summary of studies showing astrocytic-induced OS in several models of AD.

Experimental model	Prooxidative	Antioxidative	Reference
Isolated cortical astrocytes; hippocampal neurons and astrocytes in coculture, and preparations of astrocytes from rat hippocampus (Aβ_25-35_, Aβ_1-42_).	↑NOX; mitochondrial depolarization; ↑ROS	↓GSH	Abramov et al., 2004a
Primary culture of cortical rat astrocytes (Aβ_1-42_).	↑NOX; ↑ROS; mitochondrial depolarization; ↑iNOS	↓Cu/Zn SOD; =MnSOD	Hu et al., 1998; Zhu et al., 2006; Askarova et al., 2011; Bungart et al., 2014; Aguirre-Rueda et al., 2015
Mixed cultures of rat hippocampal neurons and astrocytes. Isolated cortical astrocytes (Aβ_25-35_, Aβ_1-42_).		↓GSH	Abramov et al., 2003
Cultured cortical rat astrocytes (fibrillar, oligomeric, and scrambled Aβ_1-42_).	↑iNOS and NO-derived peroxynitrite		Akama et al., 1998; Hu et al., 1998
Postmortem brains from AD patients. APP23 transgenic mice. APP induced by electrolytic cortical lesion.	↑eNOS and iNOS		Lüth et al., 2001
AD postmortem brains. nNOS immunocytochemistry in reactive astrocytes.	↑nNOS		Simic et al., 2000
Primary astrocyte culture derived from C57BL/6 (Aβ_1-42_) and derived from 5xFAD mice.		Acute ↑GSH after monomeric Aβ. Induction of ABCC1 was reduced in 6-month old 5xFAD mice.	Ye et al., 2015
Intrahippocampal injection of Aβ_25-35_. GFAP measured by ELISA in rats.	=iNOS	↓Catalase	Sohanaki et al., 2016
Cultured cortical rat astrocytes (Aβ_25-35_).	↑NOX	↓Catalase; ↓SOD, ↓GSH; ↑GST	Jeong et al., 2005
Isolated rat astrocyte cultures (Aβ_25-35_).	↑iNOS; ↑ROS; ↑RNS	↑MnSOD	Ayasolla et al., 2004
Mixed cultures of mice neurons and astrocytes. Isolated mice astrocytes (Aβ_25-35_; Aβ_1-42_).	↑ROS; ↑RNS	↓SOD; ↓GSH; ↓Catalase	Masilamoni et al., 2005b
AD postmortem human cerebral cortex and hippocampus. Immunocytochemistry for GFAP.		↑MnSOD; ↓Cu/Zn SOD	Furuta et al., 1995; Maeda et al., 1997

*ROS, reactive oxygen species; RNS, reactive nitrogen species; iNOS, inducible nitric oxide synthase; eNOS, endothelial nitric oxide synthase; nNOS, neuronal form of nitric oxide synthase; GSH, glutathione, GST, glutathione-*S*-transferase; GFAP, glial fibrillary acidic protein.*

**FIGURE 1:**
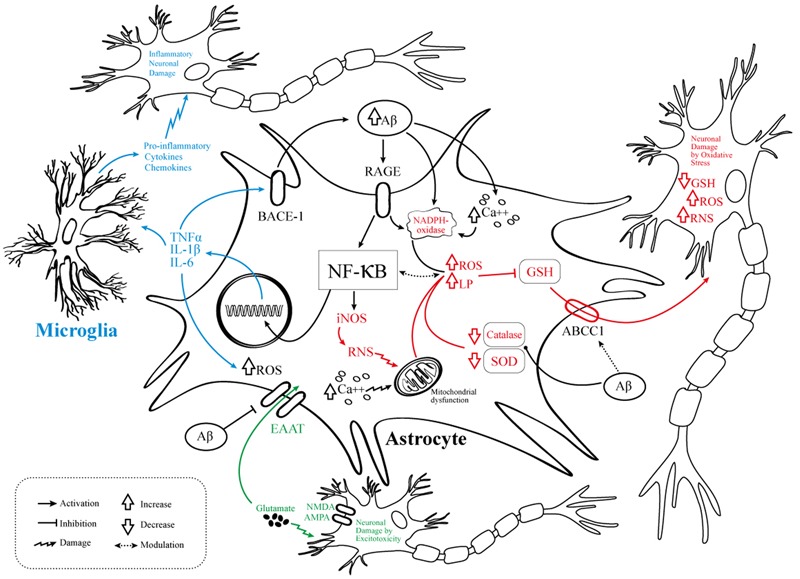
Pathophysiological events involving astrocytes in AD. Schematic representation of the molecular mechanisms linking the NF-κB pathway activation to AD pathogenesis. OS, abnormal neuroinflammatory response, and excitotoxic neuronal damage are related to several pathways of astrocyte dysfunction. In black are the elements common to the three mechanisms, namely the Aβ/RAGE/NF-kB interaction. In blue are the elements related to neuroinflammation. In red the elements related to OS. In green the elements related to neurotoxicity/excitotoxicity. Aβ, amyloid-beta; RAGE, receptor for advanced glycation products; NF-κB, nuclear factor kappa-light-chain-enhancer of activated B cells; iNOS, inducible nitric oxide synthase; RNS, reactive nitrogen species, ROS, reactive oxygen species; LP, lipid peroxidation; TNFα, tumor necrosis factor alpha; IL, interleukin; GSH, glutathione; SOD, superoxide dismutase; EAAT, excitatory amino acid transporter; BACE1, beta-secretase 1; NMDA, *N*-methyl-D-aspartate receptor; AMPA, α-amino-3-hydroxy-5-methyl-4-isoxazolepropionic acid; NADPH oxidase, nicotinamide adenine dinucleotide phosphate oxidase; Ca^++^, calcium; ABCC1, ATP-binding cassette subfamily C member 1.

Novel anti-Alzheimer’s drugs will need to consider the selective modulation of astrocyte activity in order to reduce pro-inflammatory signaling as well as to attenuate OS and diminish excitotoxicity (**Figure 1**). Taking into account the complex physiopathology of AD, a deep knowledge about dysfunctional astrocyte intracellular pathways evoked by Aβ opens the possibility for the design of new effective multi-target directed drugs.

## Novel Neuroprotective and Therapeutic Measures in Alzheimer’s Disease

Alzheimer’s disease is a major neurodegenerative disease affecting millions worldwide without a known curative treatment. Currently, only five drugs have been approved by the Food and Drug Administration (FDA) of the United States, three cholinesterase inhibitors (donepezil, galantamine, and rivastigmine), an NMDA receptor antagonist (memantine) and a combined donepezil–memantine drug. The cholinesterase inhibitors are approved for symptomatic treatment of mild-to-moderate stages of AD, while the NMDA antagonist is used for moderate-to-late stages. Regrettably none of these drugs is able to halt the progression of the disease and its uses are aimed at maximizing the quality of life of patients though broad symptom management (Caselli et al., 2017). Is therefore of great importance to design and develop new treatments which offer better therapeutic outcomes and disease-modifying responses to the patients with AD. Epidemiologic studies indicated that prolonged treatment with anti-inflammatory agents such as non-steroidal anti-inflammatory drugs could delay AD onset, as well as reduce disease rate progression (McGeer et al., 1990; Pasinetti, 2002; Cudaback et al., 2014). The inhibition of COX-mediated signaling pathways may reduce some inflammatory cytokines related with the physiopathology of AD. In addition, human studies confirm that OS plays a main role in the physiopathology of AD (Schrag et al., 2013; Chang et al., 2014). However, there are some controversies between observational studies and randomized controlled trials about the efficiency of antioxidative agents and anti-inflammatory drugs to reduce AD risk (Viña et al., 2004; Cudaback et al., 2014; Wang et al., 2015; Jiang et al., 2016). Many research groups and pharmaceutical companies have been developing new strategies to overcome the disease, but so far none of the Aβ-targeted phase three clinical trials reported has shown statistically significant benefit on its pre-specified clinical endpoints (Selkoe and Hardy, 2016). Many explanations may be offered for this lack of success, ranging from poorly designed trials to late interventions (irreversible modification of the disease due to advanced stage) but also, to the incomplete knowledge about the basic pathophysiological mechanisms of AD. As astrocytes have been shown to be involved in a diverse range of pathological changes observed in AD, they have been proposed as an interesting novel therapeutic target (Finsterwald et al., 2015). Because increased proinflammatory cytokines induced by Aβ are associated with enhanced production of free radicals in the astrocytes (Masilamoni et al., 2005a), new compounds with antioxidative and anti-inflammatory properties could reduce the effects of neurodegeneration in AD.

Various approaches involving astrocytes have been reported recently and some of them offer promising results in *in vitro*, animal, and preclinical models for the treatment of AD. In primary cortical rat astrocytes, the use of a light-generating nanoparticle attenuated Aβ-induced OS and inflammatory responses, through a reduction in the superoxide anion production and a lowering of IL-1β and iNOS expression (Bungart et al., 2014). Curcumin, a natural phenol obtained from plants and commonly used as a spice, has been proposed to be of benefit in AD, reducing Aβ formation and decreasing neurotoxicity in the brain (Lim et al., 2001; Yang et al., 2005; Cole et al., 2007). In a recent study using APP/PS1 transgenic mice and primary rat mixed neuronal/glial cultures, curcumin was reported to improve spatial memory deficits and promote cholinergic neuronal function *in vivo*, and *in vitro*, attenuated the inflammatory response of both microglia and astrocytes, acting through PPARγ, which inhibited the NF-κB signaling pathway in these cells (Liu et al., 2016). Activation of PPARγ with the use of the isoflavone phytoestrogen genistein showed an increase in the release of ApoE from primary astrocytes in an *in vivo* mouse model of AD (Bonet-Costa et al., 2016). In the same paper, the authors reported that treatment with genistein improved several cognitive features (hippocampal learning, recognition memory, implicit memory, and odor discrimination) as well as a reduction in the number and area of Aβ plaques. Neuroesteroids, such as progesterone, have been proposed to offer neuroprotection in neurodegenerative diseases including AD (Liu et al., 2013). In primary cultures of rat astrocytes, treatment with progesterone reduced Aβ-induced inflammatory responses (decreasing the production of IL-1β and TNFα), and also suppressed endoplasmic reticulum stress activation together with attenuation of PERK/elF2a signaling (Hong et al., 2016). In addition to polyphenols, many other natural phytochemicals have shown anti-inflammatory and immunosuppressive efficacy in AD models. For example, triptolide extract inhibit activation of microglia and astrocytes in the APP/PS1 transgenic mouse model of AD (Li et al., 2016). Punicalagin, a compound derived from pomegranate, not only may reduce neuroinflammation (lowering TNFα and ILβ) but also prevents OS through the reduction of iNOS, COX-2, and the production of ROS (Kim et al., 2017).

Other compounds with anti-amyloidogenic, antioxidative, and anti-inflammatory effects may have a potential role against dementia (Libro et al., 2016). For instance, the cannabinoid agonist WIN 55,212-2 has shown anti-inflammatory actions in primary cultured astrocytes after Aβ_1-42_ exposure. WIN pre-treatment prevented IL-1β, TNFα, and iNOS increase induced by Aβ. In addition, pretreatment with WIN prevented a decrease in copper/zinc-SOD induced by Aβ_1-42_ (Aguirre-Rueda et al., 2015). Cannabinoid receptor type 2 (CB2) agonist (MDA7) also reduced inflammation and also promoted clearance of amyloid plaques in the transgenic APP/PS1 mice model of AD (Wu et al., 2017). Astroglial hemichannel activity and inflammatory reactions evoked by Aβ_25-35_ were prevented by several cannabinoid receptor agonists such as WIN, 2-AG, and methanandamide (Gajardo-Gómez et al., 2017). Pantethine (B5 vitamine precursor) was able to modulate the astrocytic metabolic changes and inflammatory patterns induced by Aβ_1-42_ in astrocytes derived from the 5xFAD transgenic mouse model of AD (van Gijsel-Bonnello et al., 2017). In cultured cortical astrocytes, donepezil was shown to reduce inflammatory responses via nAChR and PI3K-Akt pathway, and to decrease intracellular ROS levels (Makitani et al., 2017). As mentioned early, donepezil is a cholinesterase inhibitor commonly used in AD patients.

Other strategies to reduce OS in AD models involve enhancers of antioxidative endogenous factors. For instance, pelargonidin (estrogen receptor agonist) increases catalase activity, reduces astrocyte activation in the hippocampus after Aβ_25-35_ exposure, and also prevents Aβ-induced spatial memory impairment (Sohanaki et al., 2016). Bambusae concretio Salicea treatment increases GSH-*S*-transferase and prevented catalase, SOD, GPX reduction, induced by Aβ_25-35_ in cultured astrocyte cells (Jeong et al., 2005). The novel compound Monascin is able to activate the expression of several antioxidative genes such as SOD-1, SOD-2, SOD-3, and Hsp16.2, and as a consequence reduced Aβ toxicity (Shi et al., 2016). *In vitro* and *in vivo* studies with exogenous antioxidative compounds such as resveratrol (Wang G. et al., 2016), tocotrienol (vitamin E form) (Ibrahim et al., 2017), anthocyanins (Rehman et al., 2017), and epicatechin (Cuevas et al., 2009) have shown beneficial effects in AD models. Finally, 3H-1,2-dithiole-3-thione, a potent free radical scavenger, is able to reduce ROS production in the AD cellular model N2a/APPswe (Wang et al., 2017).

## Conclusion

Neuroinflammation and OS are part of the functional changes frequently observed in the brains of individuals with AD. Aβ has been shown to alter the normal dynamics of both inflammatory and antioxidant and prooxidant balance, promoting an unhealthy state for the brain and neuronal–glial communication networks. Astrocytes are involved in both inflammation and OS regulation in the CNS, and seem to have a central role in the basic pathophysiological aspects that surround this neurodegenerative disease. Although the precise relation between neuroinflammation, OS, astrocytes, and AD is still not clear, the evidence points toward an important participative role of the Aβ/NF-κB interaction in astrocytes as a critical agent in the pathological mechanism of AD. Despite the continuous efforts to develop a successful treatment for AD, there is still a gap in the knowledge of the precise etiological aspects of this disease which difficult the advance of therapeutics. Therefore, and due to the evidence presented in this review, is important to start considering astrocytes as a valuable novel therapeutic and neuroprotective target for future studies related to the treatment and mechanistic comprehension of AD.

## Author Contributions

RG-R conceived the review paper. RG-R, MN-M, and KV-S edited the final version of the manuscript. RG-R wrote the section: Introduction. RG-R and MN-M wrote the section: Astrocytes and Alzheimer’s Disease. RG-R and KV-S wrote the section: Neuroinflammation, Alzheimer’s Disease, and Astrocytes. KV-S, DA-S, and LM-M wrote the section: Oxidative Stress, Alzheimer’s Disease, and Astrocytes. MN-M wrote the section: Novel Neuroprotective and Therapeutic Measures in Alzheimer’s Disease. RG-R, MN-M, DA-S, and LM-M were involved in the construction of the figure.

## Conflict of Interest Statement

The authors declare that the research was conducted in the absence of any commercial or financial relationships that could be construed as a potential conflict of interest. The reviewer ML and handling Editor declared their shared affiliation.

## Abbreviations

Aβ: amyloid beta
AD: Alzheimer’s disease
AGE: advanced glycation end products
Aldh1L1: aldehyde dehydrogenase 1 family member L1
AMPA: α-amino-3-hydroxy-5-methyl-4-isoxazolepropionic acid
APP: amyloid precursor protein
ApoE: apolipoprotein E
BACE-1: beta-site APP cleaving enzyme 1
BBB: blood–brain barrier
CaN: calcineurin
CaSR: calcium sensing receptor
CD: cluster of differentiation
CNS: central nervous system
COX: cyclooxygenase
ECE: endothelin-converting enzyme
ERK: extracellular regulated kinase
GABA: gamma-aminobutyric acid
GFAP: glial fibrillary acid protein
GLAST: glutamate aspartate transporter
GLT-1: glutamate transporter 1
GPX: glutathione peroxidase
GSH: glutathione
HMGB1: high mobility group box-1
IDE: insulin degrading enzyme
IKBα: NF-κB inhibitor alpha
IKK: IκB kinase
IL: interleukin
iNOS: inducible nitric oxide synthase
IR: insulin receptor
INF: interferon
JNK: c-Jun N-terminal kinase
MAO: monoamine oxidase
MAPK: mitogen-activated protein kinase
MMP: matrix metalloproteinase
nAChR: nicotinic acetylcholine receptor
NADH: reduced nicotinamide-adenine dinucleotide
NADPH: nicotinamide adenine dinucleotide phosphate
NEMO: NF-κB essential modulator
NEP: neprilysin
NFAT: nuclear factor of activated T-cells
NF-κB: nuclear factor kappa-light-chain-enhancer of activated B cells
NMDA: *N*-methyl-D-aspartate
NO: nitric oxide
NOS: nitric oxide synthase
8-OHG: 8-hydroxyguanosine
OS: oxidative stress
PI3K: phosphoinositide 3-kinase
PPARγ: peroxisome proliferator-activated receptor gamma
RAGE: advanced glycation end products receptor
RNS: reactive nitrogen species
ROS: reactive oxygen species
SEC-R: serpin-enzyme complex receptor
SOD: superoxide dismutase
TGF-β: transforming growth factor beta
TLR: Toll-like receptor
TNFα: tumoral necrosis factor-alpha
TRAF: tumor necrosis receptor-associated factor
TREM2: triggering receptor expressed on myeloid cells 2
